# Syndrome de loge aiguë secondaire à une injection intraveineuse d'héroïne chez un patient toxicomane: à propos d'un cas

**DOI:** 10.11604/pamj.2015.22.349.8411

**Published:** 2015-12-11

**Authors:** Adil El Alaoui, Aliou Bah

**Affiliations:** 1 Service de Chirurgie Orthopédique du Centre Hospitalier de Chambéry, France

**Keywords:** Syndrome de loge, héroïne, aponévrotomie, compartment syndrome, heroin, fasciotomy

## Image en medicine

Il s'agit d'un patient âgé de 27 ans, connu toxicomane depuis 2008 qui a présenté un syndrome de loge aigu suite à une injection d'héroïne en intraveineuse au niveau du pli du coude du membre supérieur droit. Le patient a consulté aux urgences 6 heures après l'injection d'héroïne, l'examen clinique initial a trouvé une tuméfaction du membre supérieur droit, un œdème diffus au niveau du bras et de l'avant-bras droite et un trouble de sensibilité dans le territoire du nerf médian et cubital. Le reste de l'examen clinique était normal. Le patient est opéré au urgence, il a bénéficié d'une aponévrotomie de décharge par 2 incisions au niveau de la face antérieure du bras et l'avant-bras (A). Le changement de pansement est fait chaque 2 jours au bloc opératoire par un système à pression négative de VAC^®^ (B,C). Après 5 semaines, l’évolution était bonne on a noté une disparition du syndrome de loge (D) avec récupération de la sensibilité dans le territoire du nerf médian et cubital.

**Figure 1:**
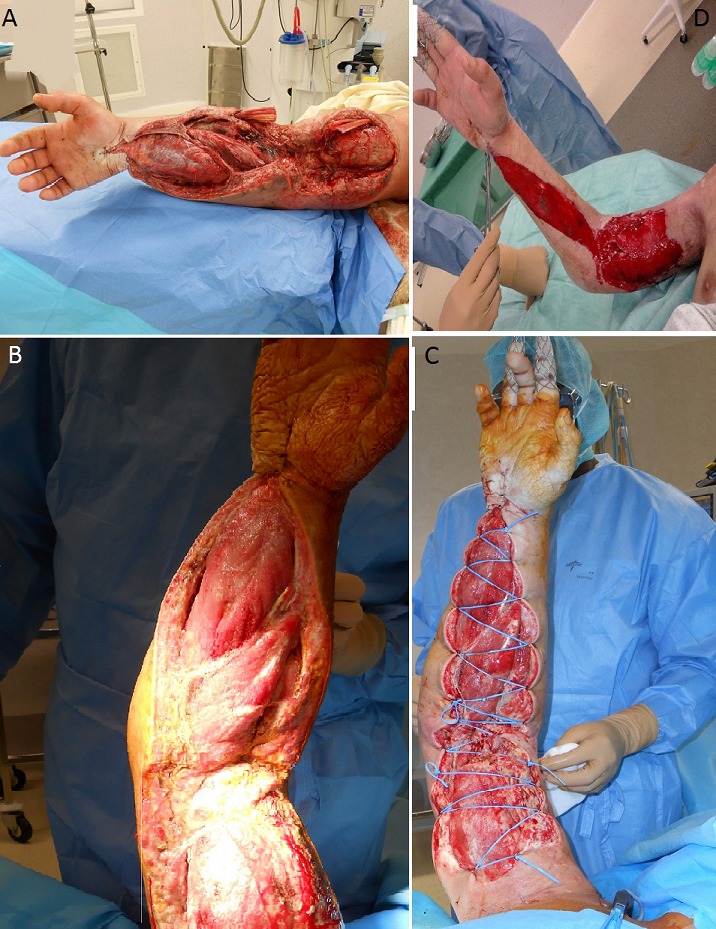
(A) image peropératoire du patient à J1 du syndrome de loge; (B) image peropératoire du patient après aponévrotomie de décharge; (C) régression progressive du syndrome de loge à J 10; (D) disparition du syndrome de loge après 5 semaines

